# Supplementary material for: The Impact of SARS-CoV-2 Infection on Heart Rate Variability: A Systematic Review of Observational Studies with Control Groups
Source: Int J Environ Res Public Health. 2023 Jan 4;20(2):909. doi: 10.3390/ijerph20020909 (PMC9859268; doi:10.3390/ijerph20020909)
Supplement: Supplementary file 1 [file ijerph-20-00909-s001.zip › ijerph-2059511-supplementary/Supplementary File S2.pdf]

Supplementary S2. Search strategies used in each

database Medline via PubMed

	Searches	Results
#1	COVID-19[MH] OR SARS-CoV-2[MH] OR COVID-19[TIAB] OR SARS-CoV-2[TIAB] OR (wuhan[TIAB] AND coronavirus[TIAB]) OR 2019-nCoV[TIAB] OR 2019nCoV[TIAB]	274,311
#2	HRV[TIAB] OR “heart rate variability”[TIAB] OR “heart rate variation”[TIAB] OR “heart beat variability”[TIAB] OR “heart beat variation”[TIAB] OR “RR variation”[TIAB]	24,840
#3	#1 AND #2	**143**

EMBASE via Elsevier

	Searches	Results
#1	‘coronavirus disease 2019’/exp OR ‘coronavirus disease 2019’:ab,ti OR COVID-19:ab,ti OR ‘Severe acute respiratory syndrome coronavirus 2’/exp OR ‘Severe acute respiratory syndrome coronavirus 2’:ab,ti OR SARS-CoV-2:ab,ti OR (wuhan:ab,ti AND coronavirus:ab,ti) OR 2019-nCoV:ab,ti OR 2019nCoV:ab,ti	308,753
#2	‘heart rate variability’/exp OR HRV:ab,ti OR ‘heart rate variability’:ab,ti OR ‘heart rate variation’:ab,ti OR ‘heart beat variability’:ab,ti OR ‘heart beat variation’:ab,ti OR ‘RR variation’:ab,ti	42,906
#3	#1 AND #2	**233**

PsycARTICLES via ProQuest

	Searches	Results
#1	SU(COVID-19) OR SU(SARS-CoV-2) OR TIAB(COVID-19) OR TIAB(SARS-CoV-2) OR (TIAB(wuhan) AND TIAB(coronavirus)) OR TIAB(2019-nCoV) OR TIAB(2019nCoV)	1,146
#2	SU(heart rate variability) OR TIAB(HRV) OR TIAB(heart rate variability) OR TIAB(heart rate variation) OR TIAB(heart beat variability) OR TIAB(heart beat variation) OR TIAB(RR variation)	233
#3	#1 AND #2	**0**

CINAHL via EBSCO

	Searches	Results
#1	COVID-19[MH] OR SARS-CoV-2[MH] OR COVID-19[TX] OR SARS-CoV-2[TX] OR (wuhan[TX] AND coronavirus[TX]) OR 2019-nCoV[TX] OR 2019nCoV[TX]	152
#2	Heart Rate Variability[MH] OR HRV[TX] heart rate variability[TX] OR heart rate variation[TX] OR heart beat variability[TX] OR heart beat variation[TX] OR RR variation [TX]	25
#3	#1 AND #2	**0**
