# Supplementary material for: The Impact of SARS-CoV-2 Infection on Heart Rate Variability: A Systematic Review of Observational Studies with Control Groups
Source: Int J Environ Res Public Health. 2023 Jan 4;20(2):909. doi: 10.3390/ijerph20020909 (PMC9859268; doi:10.3390/ijerph20020909)
Supplement: Supplementary file 1 [file ijerph-20-00909-s001.zip › ijerph-2059511-supplementary/Supplementary File S3.pdf]

Supplementary S3-1. Main findings of included studies

Author (Country)	Study type	Comparison	Main findings (unit) [direction of statistical significance]
Sari 2020 (Turkey) [49]	case-control study	G1: symptomatic COVID-19 patients (n = 25)<br>G2: asymptomatic COVID-19 patients (n = 25)<br>G3: matched controls (n = 51)	**1. infected vs. non-infected**<br>1) no significant difference ($p > 0.05$): SDNN (ms), SDANN (ms), RMSSD (ms), SDNN Index, pNN50 (%), CCVLF, CCVHF, LF/HF<br>**2. symptomatic infected vs. non-infected**<br>1) significant differences ($p < 0.05$): SDANN (ms) [<], RMSSD (ms) [<], pNN50 (%) [<], CCVHF [<], LF/HF [<]<br>2) no significant difference ($p > 0.05$): SDNN (ms), SDNN Index, CCVLF<br>**3. symptomatic vs. asymptomatic**<br>1) significant differences ($p < 0.05$): SDNN (ms) [<], SDANN (ms) [<], RMSSD (ms) [<], SDNN Index [>], pNN50 (%) [<], CCVLF [<], CCVHF [<], LF/HF [<]
Aragón-Benedí 2021 (Spain) [50]	prospective cohort study	G1: critically ill COVID-19 who survived (n = 7)<br>G2: critically ill COVID-19 who died (n = 7)	**1. survived patients vs. died patients**<br>1) significant differences ($p < 0.05$): mean ANI, index for HFnorm [<]<br>2) no significant difference ($p > 0.05$): Power (ms), index for SDNN
Bellavia 2021 (Italy) [51]	cross-sectional study	G1: COVID-19 patients (n = 20)<br>G2: COVID-19 negative controls (n = 20)	**1. infected vs. non-infected**<br>1) no significant difference ($p > 0.05$): SDNN (ms), SDANN (ms), pNN50 (%), RMSSD (ms), LF (ms^2), HF (ms^2)
Gadaleta 2021 (USA) [52]	cross-sectional study	G1: COVID-19 patients (n = 198)<br>G2: COVID-19 negative controls (n = 1614)	No relevant statistical analysis
Hirten 2021 (USA) [53]	prospective cohort study	G1: COVID-19 patients (n = 13)<br>G2: COVID-19 negative controls (n = 284)	**1. infected vs. non-infected**<br>1) significant differences ($p < 0.05$): MESOR of SDNN (ms) [>]<br>2) no significant difference ($p > 0.05$): MESOR of SDNN (ms), mean acrophase of the circadian pattern of SDNN (ms)
Junarta 2021 (USA) [54]	retrospective review	G1 (after): chronic atrial fibrillation + COVID-19 hospitalization<br>G2 (before): chronic atrial fibrillation + pre-COVID-19<br>Total N = 38	**1. infected vs. non-infected**<br>1) significant differences ($p < 0.05$): SDSD (ms) [<], RMSSD (ms) [<], pNN50 (%) [<]
Kaliyaperumal 2021 (India) [55]	case-control study	Analysis 1<br>G1: COVID-19 patients (n = 63)<br>G2: matched controls (n = 43)<br>Analysis 2<br>G3: symptomatic COVID-19 patients (n = 33)<br>G4: asymptomatic COVID-19 patients (n = 33)	**1. infected vs. non-infected**<br>1) significant differences ($p < 0.05$): HF (log data) [<], LF (ms^2) [<], RMSSD (log data) [>]<br>2) no significant difference ($p > 0.05$): HF/LF, LF/HF, pNN50 (%), SDNN (log data)<br>**2. symptomatic vs. asymptomatic**<br>1) no significant difference ($p > 0.05$): HF (log data), LF (ms^2), HF/LF, LF/HF,

			pNN50 (%), RMSSD (log data), SDNN (log data)
Kamaleswaran 2021 (USA) [56]	retrospective review	G1: critically ill COVID-19 who survived<br>G2: critically ill COVID-19 who died<br>Total N = 85	**1. survived patients vs. died patients**<br>1) significant differences ($p < 0.05$): LF/HF, VLF (ms^2), RMSSD (ms), pNN50 (%)
Khalpey 2021 (USA) [57]	retrospective review	G1: symptomatic COVID-19 patients<br>G2: asymptomatic COVID-19 patients with silent hypoxia<br>G3: asymptomatic COVID-19 negative patients with silent hypoxia<br>G4: symptomatic COVID-19 negative patients<br>Total N = 200	**1. infected vs. non-infected (G1&G2 vs. G3&G4)**<br>1) significant differences ($p < 0.05$): RMSSD (ms), SDNN (ms), HRV triangular index<br>**2. symptomatic vs. asymptomatic (G1 vs. G2)**<br>1) no significant difference ($p > 0.05$): RMSSD (ms), SDNN (ms), HRV triangular index
Lonini 2021 (USA) [58]	retrospective review	G1: COVID-19 positive patients (n = 15)<br>G2: healthy controls (n = 14)	**1. infected vs. non-infected**<br>1) significant differences ($p < 0.05$): HRV (s) [<]
Milovanovic 2021 (Serbia) [59]	case-control study	G1: mild COVID-19 patients (n = 30)<br>G2: severe COVID-19 patients (n = 45)<br>G3: matched controls (n = 77)	**1. infected, but mild vs. non-infected**<br>1) significant differences ($p < 0.05$): LF (ms^2) [<], HF (ms^2) [<]<br>2) no significant difference ($p > 0.05$): LFnorm (nu), HFnorm (nu), VLF (ms^2), LF/HFt<br>**2. infected and severe vs. non-infected**<br>1) significant differences ($p < 0.05$): LF (ms^2) [<], LF/HF [>]<br>2) no significant difference ($p > 0.05$): LFnorm (nu), HFnorm (nu), VLF (ms^2), HF (ms^2)
Pan 2021 (China) [60]	cross-sectional study	G1: mild COVID-19 patients (n = 13)<br>G2: severe COVID-19 patients (n = 21)	**1. mild vs. severe**<br>1) significant differences ($p < 0.05$): SDNN (ms) [>], SDANN (ms) [>], LF/HF [<]<br>2. no significant difference ($p > 0.05$): RMSSD (ms), pNN50 (%), LF (ms^2), HF (ms^2)
Topal 2021 (Turkey) [61]	retrospective review	G1: confirmed COVID-19 patients (n = 53)<br>G2: suspected COVID-19 patients (n = 42)<br>G3: healthy controls (n = 20)	**1. infection confirmed vs. non-infected**<br>1) significant differences ($p < 0.05$): SDNN (ms) [<], SDANN (ms) [<], RMSSD (ms) [>], NN50 count [<], HRV triangular index [<], LF (ms^2) [>], HF (ms^2) [<], LF/HF ratio [>]<br>**2. infection suspected vs. non-infected**<br>1) significant differences ($p < 0.05$): SDNN (ms) [<], SDANN (ms) [<], RMSSD (ms) [>], NN50 count [<], HRV triangular index [<], LF (ms^2) [>], HF (ms^2) [<], LF/HF ratio [>]<br>**3. infection confirmed vs. infection suspected**<br>1) significant differences ($p < 0.05$): LF (ms^2) [>], HF (ms^2) [>], LF/HF ratio [>]<br>2) no significant difference ($p > 0.05$): SDNN (ms), SDANN (ms), RMSSD (ms), NN50 count, HRV triangular index
Hirten 2022 (USA) [62]	prospective cohort study	G1: COVID-19 patients (n = 49)<br>G2: COVID-19 negative controls (n = 358)	No relevant statistical analysis

Ranard 2022 (USA) [63]	retrospective review	G1: COVID-19 patients with sudden cardiac death (n = 12)<br>G2: COVID-19 patients without sudden cardiac death (n = 18)	**1. survived patients vs. died patients**<br>1) significant differences ($p < 0.05$): RMSSD (ms) [$>$]
Risch 2022 (Switzerland) [64]	prospective cohort study	COVID-19 patients<br>G1: Baseline<br>G2: Incubation<br>G3: Presymptomatic<br>G4: Symptomatic<br>G5: Recovery<br>Total N = 66	**1. baseline vs. incubation**<br>1) significant differences ($p < 0.05$): SDNN (ms) [$>$], LF/HF [$>$]<br>2) no significant difference ($p > 0.05$): RMSSD (ms)<br>**2. baseline vs. presymptomatic**<br>1) significant differences ($p < 0.05$): SDNN (ms) [$>$], LF/HF [$>$]<br>2) no significant difference ($p > 0.05$): RMSSD (ms)<br>**3. baseline vs. symptomatic**<br>1) significant differences ($p < 0.05$): SDNN (ms) [$>$]<br>2) no significant difference ($p > 0.05$): RMSSD (ms), LF/HF<br>**3) baseline vs. recovery**<br>1) no significant difference ($p > 0.05$): SDNN (ms), RMSSD (ms), LF/HF
Skow 2022 (USA) [65]	case-control study	G1: Omicron COVID-19 patients (n = 23)<br>G2: matched controls (n = 13)	**1. infected vs. non-infected**<br>1) no significant difference ($p > 0.05$): HF (ms^2), LF (ms^2)

Abbreviations. ANI, Analgesia nociception index; CCVHF, coefficient of component variance for high frequency; CCVLF, coefficient of component variance for low frequency; COVID-19, Coronavirus disease of 2019; HF, high frequency; HFnorm, normalized unit of high frequency power; HRV, heart rate variability; LF, low frequency; LFnorm, normalized unit of low frequency power; MESOR, midline statistic of rhythm; NN, normal-to-normal; pNN50, the proportion of NN50 divided by the total number of NN intervals; RMSSD, the square root of the mean squared differences of successive NN intervals; SDANN, standard deviation of the average NN interval; SDNN, mean standard deviation of the NN interval; VLF, very low frequency.

Supplementary S3-2. Main findings on HRV parameters of included studies

HRV parameters	1. COVID-19 patients vs. negative control	1-1. COVID-19 in incubation stage vs. negative control	1-2. COVID-19 in recovery stage vs. negative control	1-3. Asymptomatic COVID-19 vs. negative control	1-4. Symptomatic COVID-19 vs. negative control	1-5. Mild COVID-19 vs. negative control	1-6. Severe COVID-19 vs. negative control	2. Symptomatic COVID-19 vs. asymptomatic COVID-19	3. Severe COVID-19 vs. mild COVID-19	4. Died COVID-19 patients vs. survived COVID-19 patients
**Time Domain Measures**										
**RMSSD (ms)**	Kaliyaperumal 2021, Topal 2021 [>]<br>Sari 2020, Bellavia 2021 [-]<br>Junarta 2021 [<]<br>Khalpey 2021 [UC]	Risch 2022 [-]	Risch 2022 [-]	Risch 2022 [-]	Risch 2022 [-]<br>Sari 2020 [<]			Khalpey 2021, Kaliyaperumal 2021 [-]<br>Sari 2020 [<]	Pan 2021 [-]	Ranard 2022 [<]<br>Kamaleswaran 2021 [UC]
**SDNN (ms)**	Sari 2020, Bellavia 2021 [-]<br>Kaliyaperumal 2021, Topal 2021 [<]<br>Khalpey 2021 [UC]	Risch 2022 [<]	Risch 2022 [-]	Risch 2022 [<]	Sari 2020 [-]<br>Risch 2022 [<]			Khalpey 2021, Kaliyaperumal 2021 [-]<br>Sari 2020 [<]	Pan 2021 [<]	Aragón-Benedí 2021 [-]
**SDNN index**	Sari 2020 [-]				Sari 2020 [-]			Sari 2020 [>]		
**mean amplitude of the circadian pattern of SDNN (ms)**	Hirten 2021 [>]									
**mean acrophase of the circadian pattern of SDNN (ms)**	Hirten 2021 [-]									
**MESOR of SDNN (ms)**	Hirten 2021 [-]									
**SDSD (ms)**	Junarta 2021 [<]									
**HRV (s)**	Lonini 2021 [<]									
**HRV triangular index**	Topal 2021 [<]<br>Khalpey 2021 [UC]							Khalpey 2021 [-]		

**NN50 count**	Topal 2021 [<]									
**pNN50 (%)**	Sari 2020, Bellavia 2021, Kaliyaperumal 2021 [-]<br>Junarta 2021 [<]				Sari 2020 [<]			Kaliyaperumal 2021 [-]<br>Sari 2020 [<]	Pan 2021 [-]	Kamaleswaran 2021 [UC]
**SDANN (ms)**	Sari 2020, Bellavia 2021 [-]<br>Topal 2021 [<]				Sari 2020 [<]			Sari 2020 [<]	Pan 2021 [<]	
**Frequency Domain Measures**										
**LF/HF**	Topal 2021 [>]<br>Sari 2020, Kaliyaperumal 2021 [-]	Risch 2022 [<]	Risch 2022 [-]	Risch 2022 [<]	Risch 2022 [-]<br>Sari 2020 [<]	Milovanovic 2021 [-]	Milovanovic 2021 [>]	Kaliyaperumal 2021 [-]<br>Sari 2020 [<]	Pan 2021 [>]	Kamaleswaran 2021 [UC]
**HF (ms^2)**	Sari 2020, Bellavia 2021, Skow 2022 [-]<br>Kaliyaperumal 2021, Topal 2021 [<]				Sari 2020 [<]	Milovanovic 2021 [<]	Milovanovic 2021 [-]	Kaliyaperumal 2021 [-]<br>Sari 2020 [<]	Pan 2021 [-]	
**HFnorm (nu)**						Milovanovic 2021 [-]	Milovanovic 2021 [-]			Aragón-Benedí 2021 [>]
**LF (ms^2)**	Topal 2021 [>]<br>Sari 2020, Bellavia 2021, Skow 2022 [-]<br>Kaliyaperumal 2021 [<]				Sari 2020 [-]	Milovanovic 2021 [<]	Milovanovic 2021 [<]	Kaliyaperumal 2021 [-]<br>Sari 2020 [<]	Pan 2021 [-]	
**LFnorm (nu)**						Milovanovic 2021 [-]	Milovanovic 2021 [-]			
**VLF (ms^2)**						Milovanovic 2021 [-]	Milovanovic 2021 [-]			Kamaleswaran 2021 [UC]

Abbreviations. COVID-19, Coronavirus disease of 2019; HF, high frequency; HFnorm, normalized unit of high frequency power; HRV, heart rate variability; LF, low frequency; LFnorm, normalized unit of low frequency power; MESOR, midline statistic of rhythm; NN, normal-to-normal; pNN50, the proportion of NN50 divided by the total number of NN intervals; RMSSD, the square root of the mean squared differences of successive NN intervals; SDANN, standard deviation of the average NN interval; SDNN, mean standard deviation of the NN interval; UC, unclear; VLF, very low frequency. **Note.** Yellow cells indicate that there is no statistically significant difference, red cells indicate that the latter in the comparison is statistically significantly larger, green cells indicate that the former in the comparison is statistically significantly greater, and gray cells indicate that there is a statistically significant difference reported, but the direction was not reported in the study.
