# Supplementary material for: The Impact of SARS-CoV-2 Infection on Heart Rate Variability: A Systematic Review of Observational Studies with Control Groups
Source: Int J Environ Res Public Health. 2023 Jan 4;20(2):909. doi: 10.3390/ijerph20020909 (PMC9859268; doi:10.3390/ijerph20020909)

Supplementary S4. Forest plot to investigate the heterogeneity of included studies

1. COVID-19 positive patients vs. negative controls

1) Vagally mediated HRV parameters

(1) RMSSD (ms)

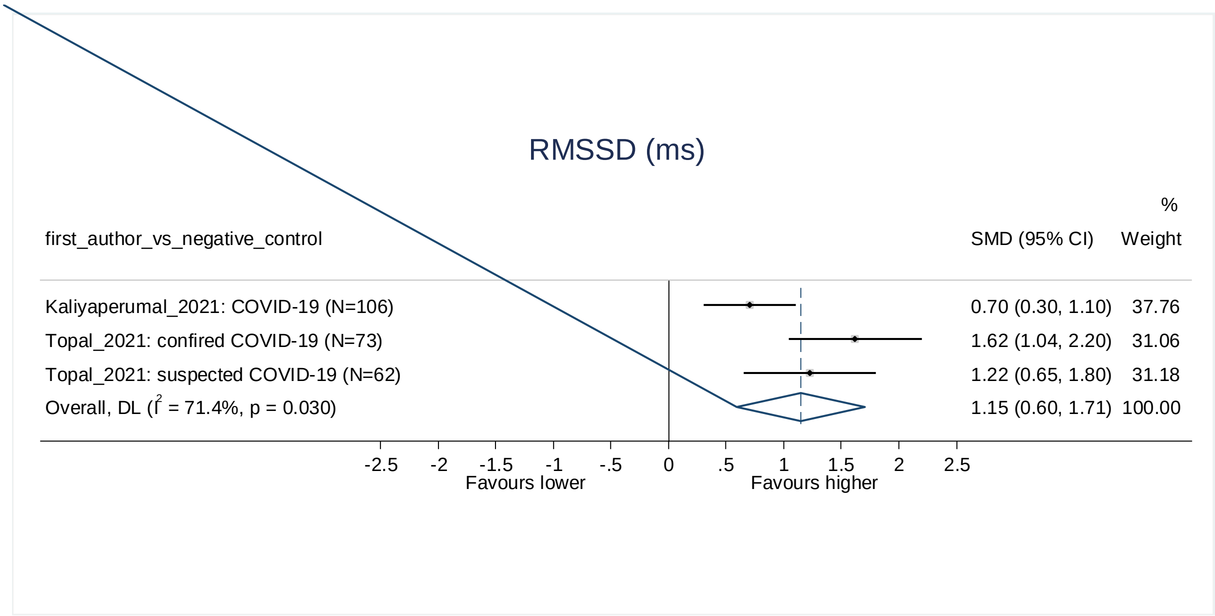

(2) HF power (ms^2)

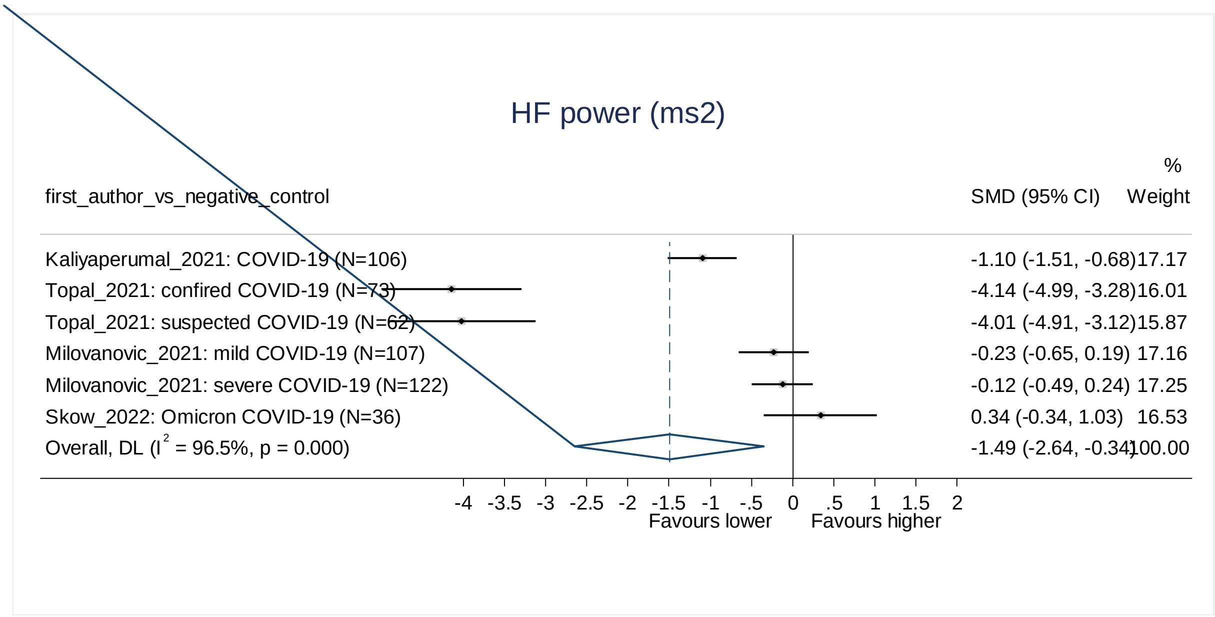

2) Other HRV parameters

(1) SDNN (ms)

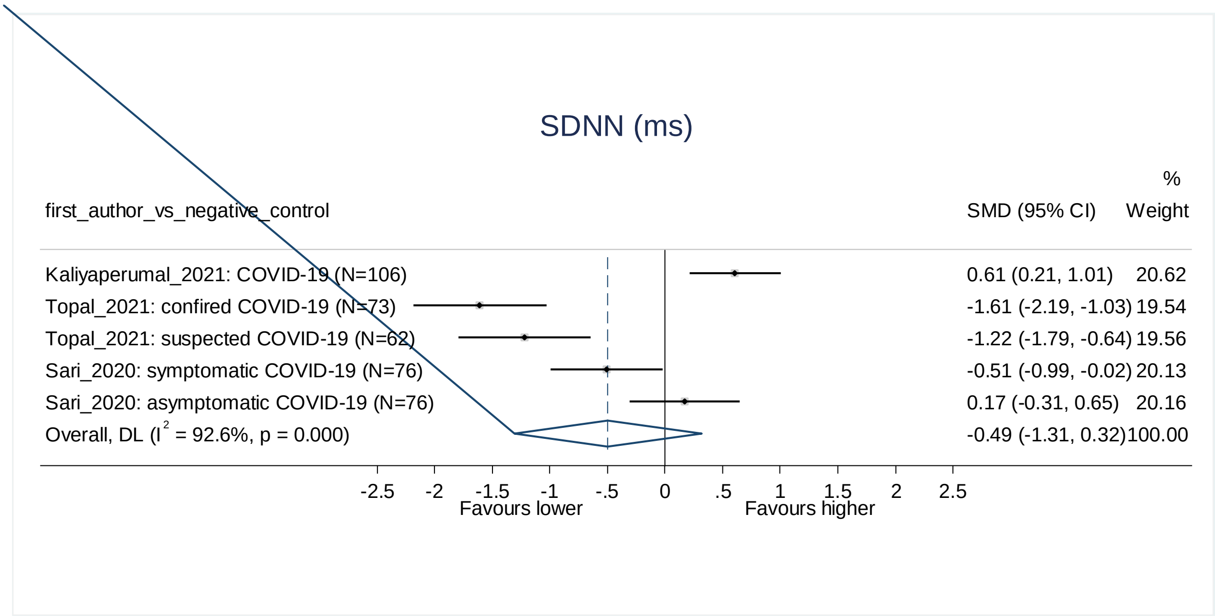

(2) LF power (ms^2)

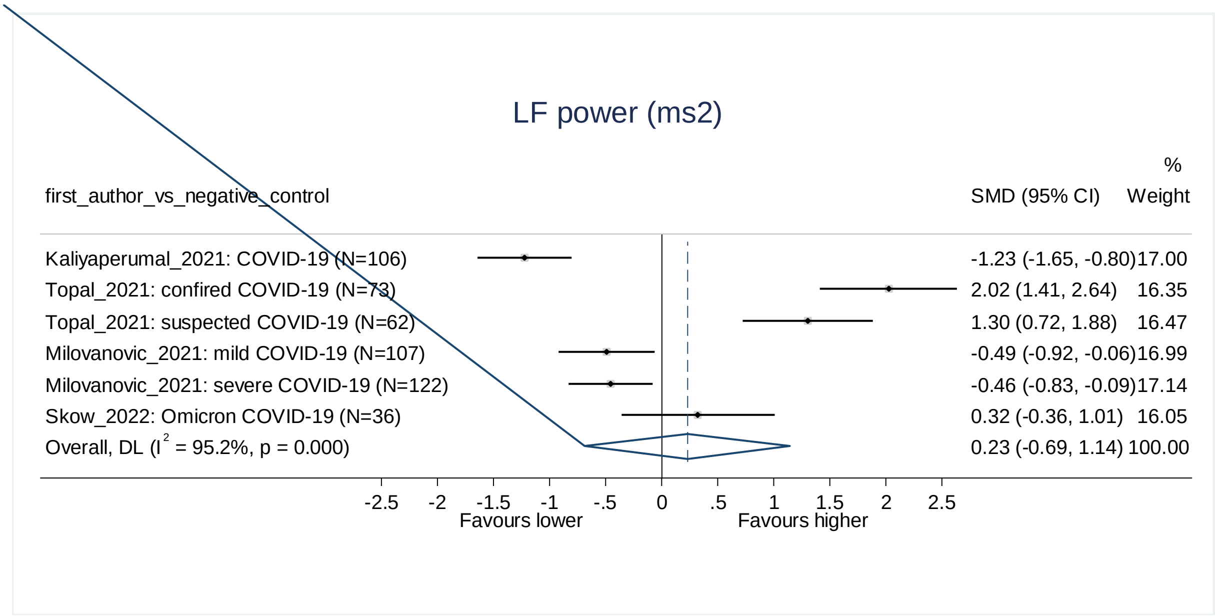

(3) LF/HF ratio

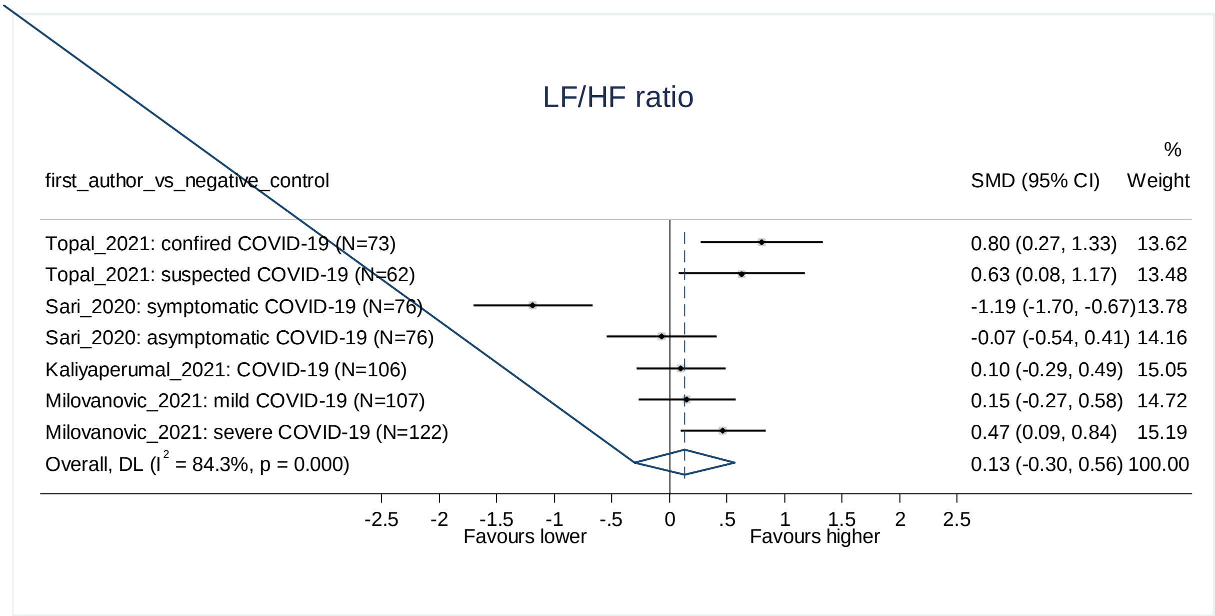

LF/HF ratio
%
first_author_vs_negative_control
SMD (95% CI)
Weight
Topal_2021: confired COVID-19 (N=73)
0.80 (0.27, 1.33)
13.62
Topal_2021: suspected COVID-19 (N=62)
0.63 (0.08, 1.17)
13.48
Sari_2020: symptomatic COVID-19 (N=76)
-1.19 (-1.70, -0.67)
13.78
Sari_2020: asymptomatic COVID-19 (N=76)
-0.07 (-0.54, 0.41)
14.16
Kaliyaperumal_2021: COVID-19 (N=106)
0.10 (-0.29, 0.49)
15.05
Milovanovic_2021: mild COVID-19 (N=107)
0.15 (-0.27, 0.58)
14.72
Milovanovic_2021: severe COVID-19 (N=122)
0.47 (0.09, 0.84)
15.19
Overall, DL (I2 = 84.3%, p = 0.000)
0.13 (-0.30, 0.56)
100.00
-2.5
-2
-1.5
-1
-.5
0
.5
1
1.5
2
2.5
Favours lower
Favours higher

2. Symptomatic COVID-19 patients vs. asymptomatic controls

(1) SDNN (ms)

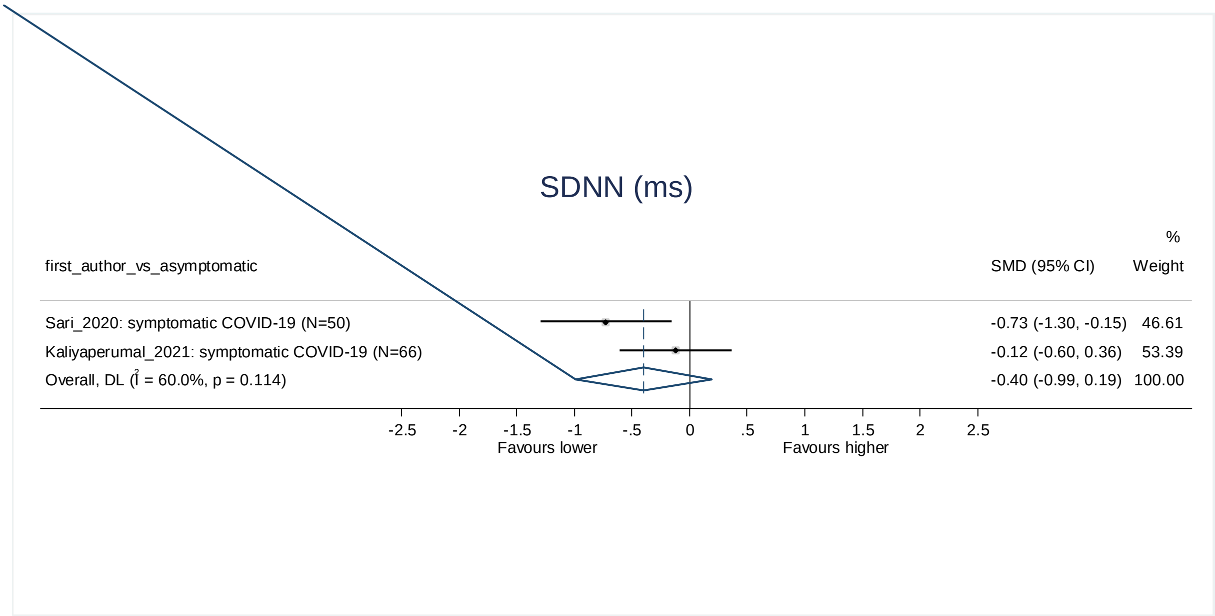

(2) LF/HF ratio

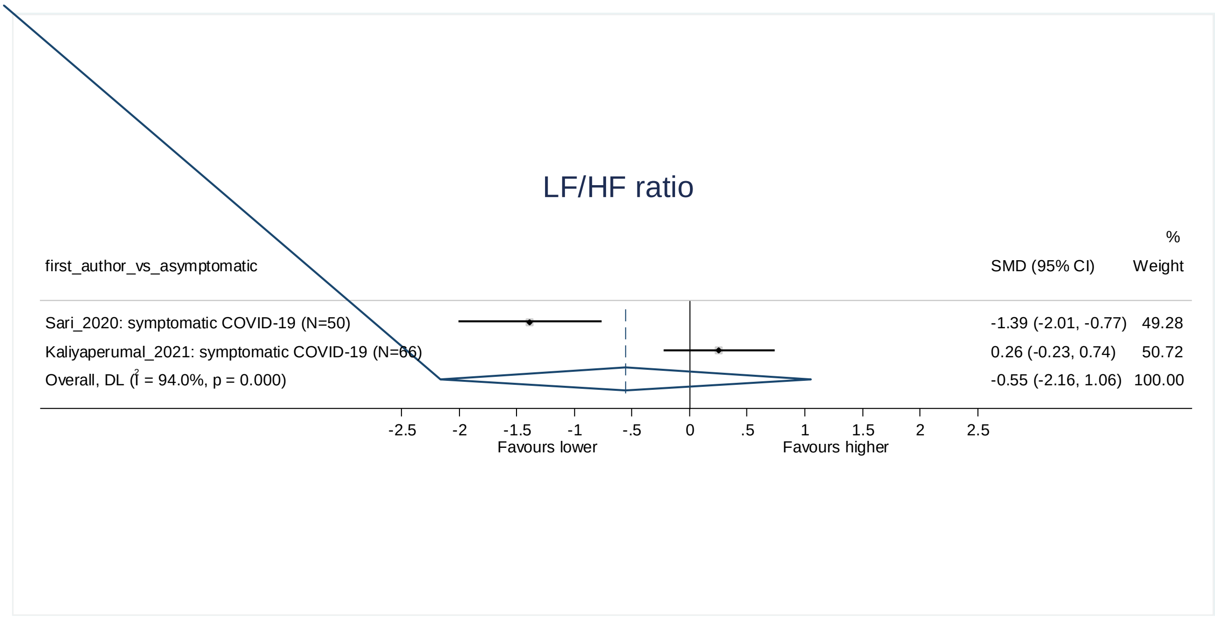

Supplement: Supplementary file 1 [file ijerph-20-00909-s001.zip › ijerph-2059511-supplementary/Supplementary File S4.pdf]
