# Supplementary material for: The Impact of SARS-CoV-2 Infection on Heart Rate Variability: A Systematic Review of Observational Studies with Control Groups
Source: Int J Environ Res Public Health. 2023 Jan 4;20(2):909. doi: 10.3390/ijerph20020909 (PMC9859268; doi:10.3390/ijerph20020909)
Supplement: Supplementary file 1 [file ijerph-20-00909-s001.zip › ijerph-2059511-supplementary/Supplementary File S5.pdf]

Supplementary S5. Bibliographic information of included studies

Author	Title	Journal	Volume (number)	Pages	DOI
Sari 2020 [49]	The effect of disease severity on cardiac autonomic functions in COVID-19	Anatolian Journal of Cardiology	24 (suppl 1)	13	Not reported
Aragón-Benedí 2021 [50]	Is the heart rate variability monitoring using the analgesia nociception index a predictor of illness severity and mortality in critically ill patients with COVID-19? A pilot study	PLoS One	16 (3)	e0249128	10.1371/journal.pone.0249128
Bellavia 2021 [51]	Instrumental Evaluation of COVID-19 Related Dysautonomia in Non-Critically-Ill Patients: An Observational, Cross-Sectional Study	J Clin Med	10 (24)	5861	10.3390/jcm10245861
Gadaleta 2021 [52]	Passive detection of COVID-19 with wearable sensors and explainable machine learning algorithms	NPJ Digit Med	4 (1)	166	10.1038/s41746-021-00533-1
Hirten 2021 [53]	Use of Physiological Data From a Wearable Device to Identify SARS-CoV-2 Infection and Symptoms and Predict COVID-19 Diagnosis: Observational Study	J Med Internet Res	23 (2)	e26107	10.2196/26107
Junarta 2021 [54]	Describing heart rate variability in patients with chronic atrial fibrillation during hospitalization for COVID-19	J Arrhythm	37 (4)	893-898	10.1002/joa3.12569
Kaliyaperumal 2021 [55]	Characterization of cardiac autonomic function in COVID-19 using heart rate variability: a hospital based preliminary observational study	J Basic Clin Physiol Pharmacol	32 (3)	247-253	10.1515/jbcpp-2020-0378
Kamaleswaran 2021 [56]	Altered heart rate variability predicts mortality early among critically ill COVID-19 patients	Critical Care Medicine	49 (1)	99	10.1097/01.ccm.0000726796.82178.b5
Khalpey 2021 [57]	Autonomic Dysfunction in COVID-19: Early Detection and Prediction Using Heart Rate Variability	Journal of the American College of Surgeons	233 (5)	e20-21	10.1016/j.jamcollsurg.2021.08.057
Lonini 2021 [58]	Rapid Screening of Physiological Changes Associated With COVID-19 Using Soft-Wearables and Structured Activities: A Pilot Study	IEEE J Transl Eng Health Med	9	4900311	10.1109/jtehm.2021.3058841
Milovanovic 2021 [59]	Assessment of Autonomic Nervous System Dysfunction in the Early Phase of Infection With SARS-CoV-2 Virus	Front Neurosci	15	640835	10.3389/fnins.2021.640835
Pan 2021 [60]	Alteration of Autonomic Nervous System Is Associated With Severity and Outcomes in Patients With COVID-19	Front Physiol	12	630038	10.3389/fphys.2021.630038
Topal 2021 [61]	Heart Rate Variability in Hospitalized Patients with Suspected or Confirmed Diagnosis of COVID-19: A Retrospective Analysis in	Celal Bayar Üniversitesi	8 (3)	516-523	10.34087/cbusbed.983215

	Comparison to Healthy Controls and in Relation to Proinflammatory Cytokines	Sağlık Bilimleri Enstitüsü Dergisi			
Hirten 2022 [62]	Evaluation of a machine learning approach utilizing wearable data for prediction of SARS-CoV-2 infection in healthcare workers	JAMIA Open	5 (2)	ooac041	10.1093/jamiaopen/ooac041
Ranard 2022 [63]	Heart rate variability and adrenal size provide clues to sudden cardiac death in hospitalized COVID-19 patients	J Crit Care	71	154114	10.1016/j.jcrc.2022.154114
Risch 2022 [64]	Investigation of the use of a sensor bracelet for the presymptomatic detection of changes in physiological parameters related to COVID-19: an interim analysis of a prospective cohort study (COVI-GAPP)	BMJ Open	12 (6)	e058274	10.1136/bmjopen-2021-058274
Skow 2022 [65]	Impact of breakthrough COVID-19 cases during the omicron wave on vascular health and cardiac autonomic function in young adults	Am J Physiol Heart Circ Physiol	323 (1)	h59-64	10.1152/ajpheart.00189.2022
